# Global & Community Health: What Did the COVID-19 Pandemic Teach Us About Neurologic Surveillance Approaches, and How Should We Be Better Prepared?

**DOI:** 10.1212/WNL.0000000000214431

**Published:** 2025-11-24

**Authors:** Rachael Matthews, Mark Alexander Ellul, Stephen McKeever, Thomas Pollack, Catherine Houlihan, Kiran Teresa Thakur, Sherry Hsiang-Yi Chou, Jennifer A. Frontera, Deanna R. Saylor, Mashina Chomba, Elena Moro, Stephen T.J. Ray, Malcolm G. Semple, Craig J. Smith, Martin R. Turner, Edward Bullmore, Alan Carson, Iain Buchan, Gerome Breen, Tom Solomon, Timothy R. Nicholson, Sarah Pett, Rhys H. Thomas, Benedict Daniel Michael

**Affiliations:** 1 Clinical Infection, Microbiology, and Immunology, Institute of Infection, Veterinary and Ecological Sciences, University of Liverpool, United Kingdom;; 2 The Walton Centre NHS Foundation Trust, Liverpool, United Kingdom;; 3 Department of Psychosis Studies, Institute of Psychiatry, Psychology and Neuroscience, King's College London, United Kingdom;; 4 Rare and Imported Pathogens Laboratory (RIPL), Porton Down, United Kingdom;; 5 Program in Neuroinfectious Diseases, Department of Neurology, Columbia University Irving Medical Center-New York Presbyterian Hospital, New York;; 6 Department of Neurology, Northwestern University Feinberg School of Medicine, Chicago, IL;; 7 Department of Neurology, NYU Grossman School of Medicine, New York;; 8 Department of Neurology, Johns Hopkins University School of Medicine, Baltimore, MD;; 9 Department of Medicine, Neurology Division, University Teaching Hospital, Lusaka, Zambia;; 10 Grenoble Alpes University, Centre Hospitalier Universitaire of Grenoble, Grenoble Institute of Neuroscience, INSERM, France;; 11 Oxford Vaccine Group, Department of Paediatrics, University of Oxford, United Kingdom;; 12 Manchester Centre for Clinical Neurosciences, Geoffrey Jefferson Brain Research Centre, Manchester Academic Health Science Centre, Salford Royal Foundation Trust, Salford, United Kingdom;; 13 Division of Cardiovascular Sciences, University of Manchester, Oxford Road, Manchester;; 14 Nuffield Department of Clinical Neurosciences, University of Oxford, United Kingdom;; 15 Department of Psychiatry, University of Cambridge, United Kingdom;; 16 Wolfson Brain Imaging Centre, Department of Clinical Neurosciences, University of Cambridge, United Kingdom;; 17 Centre for Clinical Brain Sciences, University of Edinburgh, United Kingdom;; 18 Institute of Population Health, University of Liverpool, United Kingdom;; 19 Medical Research Council Clinical Trials Unit, Institute of Clinical Trials and Methodology, University College London, United Kingdom;; 20 Department of Neurology, Royal Victoria Infirmary, Newcastle, United Kingdom; and; 21 Translational and Clinical Research Institute, Newcastle University, United Kingdom.

## Abstract

It is well recognized that many pandemic viruses are associated with neurologic complications, most recently with COVID-19. After the outbreak of the COVID-19 pandemic, neurologic surveillance platforms were implemented to characterize the complications of COVID-19. Surveillance platforms are invaluable in providing timely data, informing clinical practice, and directing future research. Lessons learned from recent neurologic surveillance networks include the importance of global and cross-specialty collaboration. It is critical for future surveillance systems to consider these aspects, as it will also serve to improve representation of low and middle-income countries (LMICs) and communities. Trainees played a critical role in the success of neurologic surveillance networks; as frontline health care workers, they were able to provide timely data collection, and their fresh insights are important for future pandemic surveillance system development. In this article, we review the methods of recent neurologic surveillance networks and discuss their strengths and limitations. We explore the outlook for pandemic surveillance platforms and the crucial role global collaboration plays in ensuring that LMICs are represented. We review the role of trainees in pandemic surveillance networks and discuss how it is vital to encourage their continued involvement to ensure that, as future health care leaders, they are prepared to manage future pandemics effectively.

## Introduction

It has become increasingly recognized that many pandemic respiratory viruses are associated with acute and postinfectious effects on the nervous system, most recently with COVID-19.^1^ During the initial phase of the COVID-19 pandemic, numerous published reports of associated neurologic complications prompted implementation of neurologic surveillance networks to investigate the breadth of complications of COVID-19.^2^

Neurologic surveillance platforms are essential for timely identification of emerging neurologic patterns of disease and enable clinicians, scientists, and public health authorities to inform clinical care, policy, and research. A successful surveillance system requires input from multiple disciplines, including clinicians, epidemiologists, and data scientists, to develop common clinical case definitions and classifications, plan study designs, and perform analytical modeling. Digital systems must be secure, communicate and share data with other systems, and be easy for users to understand and operate. National and international health organizations in addition to professional bodies play a vital role in governance, ethical considerations, data standardization, and global collaboration.

## Surveillance Studies to Date

We describe 3 simultaneous and independent neurologic surveillance efforts, which were adopted in the United Kingdom, the United States, and Europe during the COVID-19 pandemic. The Table summarizes the strengths and weaknesses of each effort.

**Table:** Summary of Strengths and Weaknesses of the Different Neurologic Surveillance Platforms Adopted During the COVID-19 Pandemic

Neurologic surveillance platform	Strengths	Weaknesses
CoroNerve	•Collected close to real-time data •Could be completed using a phone •Involved multiple neurosciences allied specialists •Regular multidisciplinary team meetings to discuss complex cases •Transparent publication policy	•Restricted to 1 country •Focus on hospitalized patients •Lack of preexisting communication channels between neuroscience bodies
ENERGY	•Used the existing EAN network of neurologists •Involved multiple European and non-European countries •Examinations were completed by a neurologist •Prospective 5-y follow-up	•Focus on hospitalized patients •Follow-up is made by phone call
GCS-NeuroCOVID	•Involved multiple countries and continents •Pragmatic design prioritizing feasibility allowed rapid ramp-up of participating sites •Able to capture a “snap shot in time” of neurologic manifestations during the pandemic's most intense periods •Developed common data elements and standardized data forms available in English, Spanish, and Portuguese	•Focus on hospitalized patients •Limited long-term follow-up data •Minimal funding support limited growth and long-term sustainability of the established consortium

Abbreviation: EAN = European Academy of Neurologists.

The CoroNerve Studies Group conducted a UK-wide surveillance study on the neurologic and psychiatric complications of COVID-19.^3-5^ Formed by clinicians and researchers in response to the COVID-19 outbreak, the group coordinated a call-out to representatives of each of the clinical neuroscience bodies across the United Kingdom (members of whom are consultants or specialist trainees) representing neurology, stroke, psychiatry, intensive care, and pediatrics. Reporting systems were hosted on the web platforms of these collaborating professional bodies and launched independently but in quick succession through an online network of rapid response web portals in April 2020. Clinicians were encouraged to submit cases prospectively and to allocate the case to 1 or more broad clinical syndromes, as defined in standardized case record forms (CRFs). Strengths of CoroNerve were evident in the voluntary collaboration of the various neuroscience bodies in response to the emerging pandemic, despite the lack of a preexisting central governing infrastructure. The group rapidly developed CRFs with predefined clinical syndromes to ensure standardization of data collection and ease of use for clinicians. Regular multidisciplinary meetings of key representatives took place to discuss difficult and complex syndromic diagnoses, which required allocation to specific phenotypes. In addition, there was a transparent policy regarding publication of any salient results, which gave authorship rights to all those contributing to the study. CRFs could be completed from various devices, including cellular phones for user ease and efficiency. Swifter implementation and a more unified approach could have been achieved through a preexisting communication channel between neurosciences bodies. Trainees were heavily involved in the CoroNerve surveillance system, by assisting in the development of online reporting portals, collecting and analyzing data, and earning first authorship on publications.

In the United States, the Global Consortium Study of Neurologic Dysfunction in COVID-19 (GCS-NeuroCOVID) was launched in late March 2020 as a large international, multicentered prospective cohort study. The study was coordinated by the University of Pittsburgh and collected data from March to October 2020 across 28 centers in 13 countries.^6,7^ It was developed and led by critical care clinicians associated with the Neurocritical Care Society (NCS), and individual institutions/hospitals were invited to register for participation through a centralized web portal on their website. Leveraging of existing NCS international partnerships achieved recruitment at centers outside the United States. Strengths of GCS-NeuroCOVID study include its rapid, pragmatic, and globally inclusive design, which enabled real-time data capture. The study was grounded in a practical understanding of what data could be feasibly and reliably collected under crisis conditions, resulting in a valuable “snapshot in time” of neurologic manifestations. Its modular design allowed participating centers to choose the tier of study that best matched their local resources and conditions, ensuring broad accessibility. Tier of study refers to whether research is conducted using unconsented data (Tier 0) or requires explicit participant consent (Tier 1). While local sites began data collection with support for their specific regulatory needs, central coordination teams worked to establish multisite regulatory approvals. The consortium also rapidly developed consensus-based common data elements (CDEs) and a comprehensive data dictionary, which supported standardized data collection.^8^ Areas for improvement include expanding beyond its focus on hospitalized patients to capture neurologic outcomes across the full spectrum of COVID-19 severity. In addition, limited funding support constrained the consortium's growth and long-term sustainability, underscoring the need for more robust financial investment. Trainees were able to contribute remotely by assisting with data collection through electronic health records (EHRs) and collaborating with frontline clinicians who had direct access to clinical data. Each participating site retained ownership of its data, enabling local investigators and trainees to pursue site-specific analyses and initiate independent research projects.

The European Academy of Neurologists (EAN) launched the international Neuro-COVID registry (ENERGY) in March 2020.^6^ Members of the EAN were invited to submit CRFs for patients with confirmed and suspected COVID-19 who presented with neurologic symptoms. By 2023, patients across almost 30 countries had been included in the ENERGY registry. Key strengths included examination of all patients by a neurologist, thus assuring accuracy of neurologic signs and symptoms. Moreover, ENERGY assures a prospective 5-year follow-up. ENERGY shares the same limits as GCS-NeuroCOVID study regarding focus only on hospitalized patients. Similar to other platforms discussed, trainees were involved in data collection and analysis.

GCS-NeuroCOVID collaborated with the ENERGY consortium to pool data for 3,744 patients with COVID-19. These groups recognized that rapid and parallel implementation of multiple global surveillance strategies may result in data discrepancies and errors due to lack of consensus on clinical definitions. They established a formal partnership based on complementary geographic coverage and clear harmonization of CDEs.^8,9^ Collaborating countries within each group were determined through extensive correspondence with national and regional societies and included the Latin American Brain Injury Consortium and the POSSIBLE network in Latin America. This was largely deliverable through translation of harmonized CDEs, data definition, and CRFs into Spanish and Portuguese.^9^

## Future Directions

The collective response of the scientific community, simply put, needs to be quicker, bigger, and better integrated. Future approaches should include standardization of data collection across all surveillance systems.^10^ The goal should be to establish internationally agreed-upon case definitions, alongside standardized minimum data sets to ensure comparability across surveillance systems. The need for real-time data collection should be considered, and there is the potential to integrate neurologic surveillance systems with existing platforms of disease surveillance such as World Health Organization platforms, as well as national patient EHRs. Barriers to the latter include variability in data storage repositories and EHR systems across countries, in addition to ethical considerations surrounding data sharing and security. Collaboration is essential for the success of any future surveillance networks, in respect to both across countries and among different disciplines. CoroNerve, ENERGY, and GCS-NeuroCOVID succeeded in bringing together complementary expertise from neurologists, stroke physicians, psychiatrists, acute medicine physicians, intensivists, and pediatricians. However, additional involvement of epidemiologists, virologists, immunologists, and data scientists within neurosurveillance systems would enhance effectiveness of such systems.

There is a distinct lack of community data from primary care and low and middle-income country (LMIC) data, largely reflecting resource constraints and limited infrastructure for recognizing and recording associations between infection and neurologic syndromes.^11^ Approaches to improving community and LMIC data acquisition should include community outreach to offer support and understanding of the importance of gathering information. This should be provided in a manner that is sensitive to the cultural or language constraints of the population. A practical option includes the use of data notification platforms accessible through cellular phones with minimal data and connectivity requirements, such as that used in CoroNerve. In LMICs, limited regional availability of EHRs hinders collection and collation of data. Capacity building in resource-limited countries is essential to establish local surveillance teams and systems that will inform global databases. Although, in resource-limited settings, the argument for facilitation of improved health care services may outweigh the need for local and global reporting infrastructure, concerted efforts to improve monitoring of disease outbreak in these areas will lead to greater understanding of the disease burden, informing policy makers and, therefore, priorities for the provision of health care needs. Expanding current collaborations in a global reporting infrastructure, and extending support and resources to underrepresented groups, would facilitate greater collection of comprehensive data. This would improve the ability to recognize rarer and overlapping presentations, assist in developing global standardized diagnostic criteria, and improve care for patients globally. A possible pathway to improvement would be through collaboration of the aforementioned neurosurveillance systems with the governing bodies of neuroscience in LMICs (e.g., the African Academy of Neurology).

Finally, our findings highlight the importance of trainees in the success of neurologic surveillance platforms. Interns, residents, and fellows contributed substantially by collecting data, developing online portals, and analyzing and reporting scientific findings. Their involvement in building accurate, real-time, and reliable surveillance networks enhanced their understanding of epidemiology and public health policy, while also exposing them to the value of multidisciplinary, local, and global collaboration. The teams, in turn, benefited from the fresh perspectives trainees brought. These early experiences can help strengthen trainees' future careers as clinicians, researchers, and public health advocates.

## Conclusion

Lessons learned from recent neurologic surveillance networks include the importance of global and cross-specialty collaboration. Among the strengths of the networks are their rapid deployment, collaboration between specialties and countries, development of standardized record forms, and ease of use. Limitations compromise a focus on hospitalized patients and a lack of community and LMIC involvement. Future pandemic neurologic surveillance platforms must prioritize timely data capture, collation, and curation at a global scale. Such a platform must be accessible in resource-limited as well as resource-rich settings.^12,13^ By contributing to neurologic surveillance platforms, trainees gain invaluable skills, perspectives, and collaborative experiences that will shape their future careers.
